# Data set on concentrations, bioavailability, dose and lung deposition of labile metals bound to inhalable and respirable fractions of ambient particulate matters in Akure suburbs

**DOI:** 10.1016/j.dib.2018.06.092

**Published:** 2018-07-03

**Authors:** Emmanuel Gbenga Olumayede, Ilemobayo Oguntimehin, Faraday Thompson Ediagbonya, Chekwube Ojiodu, Grace Olubunmi Sodipe

**Affiliations:** a Department of Industrial Chemistry, Federal University, Oye, Ekiti, Nigeria; b Department of Chemical Sciences, Ondo State University of Science and Technology, Okitipupa, Ondo State, Nigeria; c Department of Science Laboratory, Yaba College of Technology, Lagos, Nigeria; d Department of Animal and Environmental Biology, Federal University, Oye, Ekiti, Nigeria

**Keywords:** Ambient particulate matter, Inhalable and respirable, Metals, Health risk, Exposure dose

## Abstract

This article consists of data sets of concentrations, dose and deposition of some labile metals bound to inhale ambient particulate matter collected at human breathing height of 1.5–2 m in Akure, South Western Nigeria. Ten (10) data points, of different air quality, were selected for study using active sampling method; during the dry season months of November, 2016 to March, 2017. At each data point, the dust particles were collected four times, sorted into inhalable and respirable fractions. The metal concentrations in each fraction were determined using Perkin-Elmer 6000 Inductively Coupled Plasma - Atomic Emission Spectrometry (ICP-AES) analysis. The data set were processed and analyzed via descriptive statistics (averages and standard deviations), and numerical analyses. The data were explored further to estimate the exposure dose of metal particles and deposition in various regions of lung (alveolar, trachea-bronchial and extra thoracic) in adults (male and female) dwelling in the area. The data revealed that the highest dose and deposition of metals (Pb, Cd and Cr) occur in the alveolar region of the lung of adults.

**Specification Table**

**Table:** 

Subject area	*Environmental Science*
More specific subject area	*Air quality and Health*
Type of data	*Table and figure*
How data was acquired	*Active sampling method*
Data format	*Raw and analyzed*
Experimental factors	*Metals concentrations in Particulate matters were analyzed in atmosphere of Akure using Perkin-Elmer 6000 Inductively Coupled Plasma - Atomic Emission Spectrometry (ICP-AES) analysis*
Experimental features	*Total metal concentrations, bioavailability, dose and lung deposition*
Data source location	*Akure suburbs, Akure South Local Government, Ondo State, Nigeria*
Data Accessibility	*All data are presented in this article*

**Value of data**

• The data generated could stimulate environmental concerns on the impacts of airborne particulates
• Data in this article format can be explored in risks assessment
• The data could be translated into improved respiratory health among the people exposed to ambient particulate matters.

## 1 Data

**Fig. 1:**
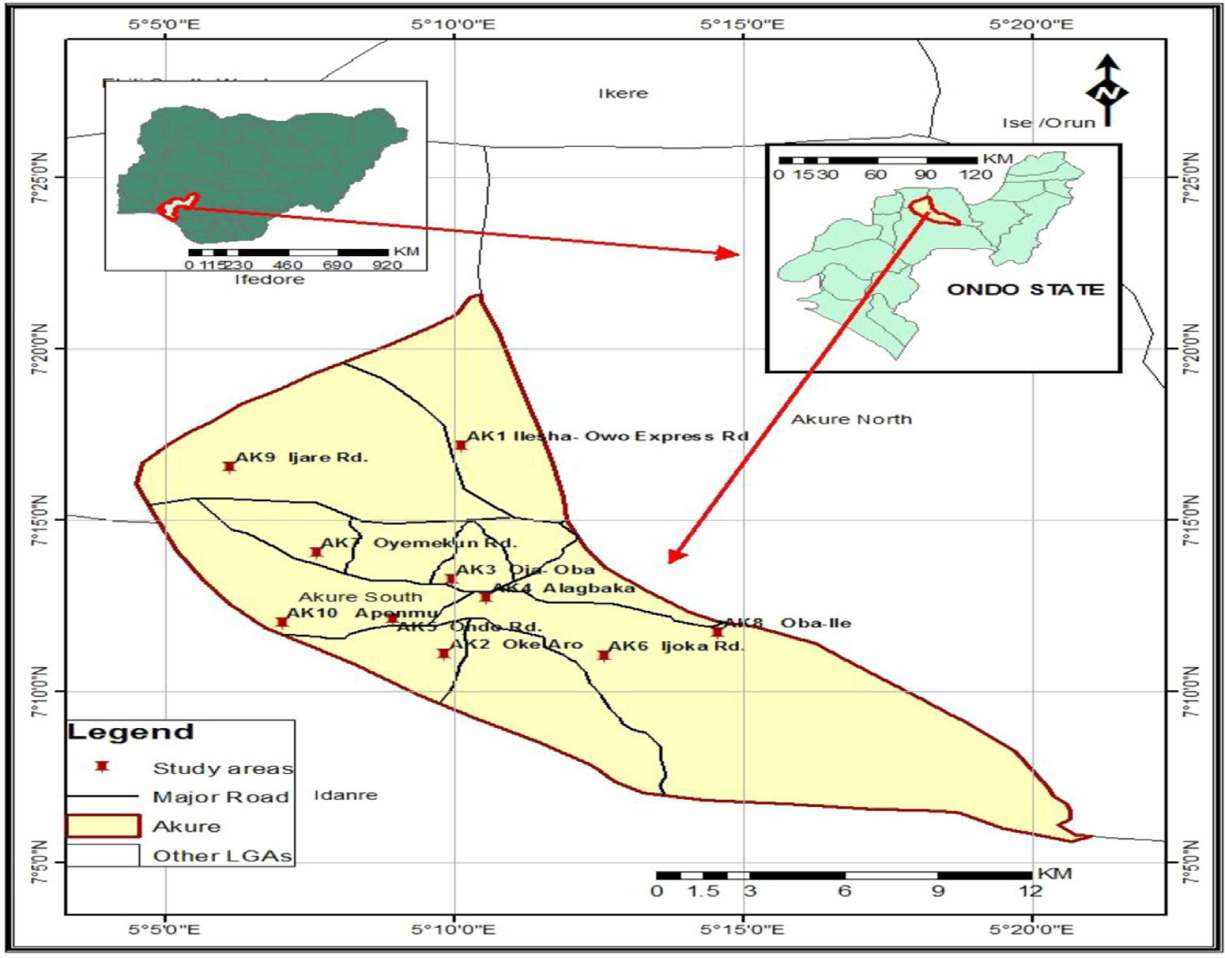
The map and location of sampling sites.

**Fig. 2:**
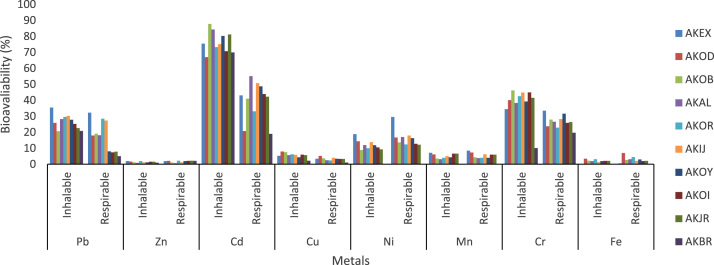
Plot of metal soluble extracted fraction (bioavailability) of the various metals using two-step physiological extraction (PBET).

**Fig. 3:**
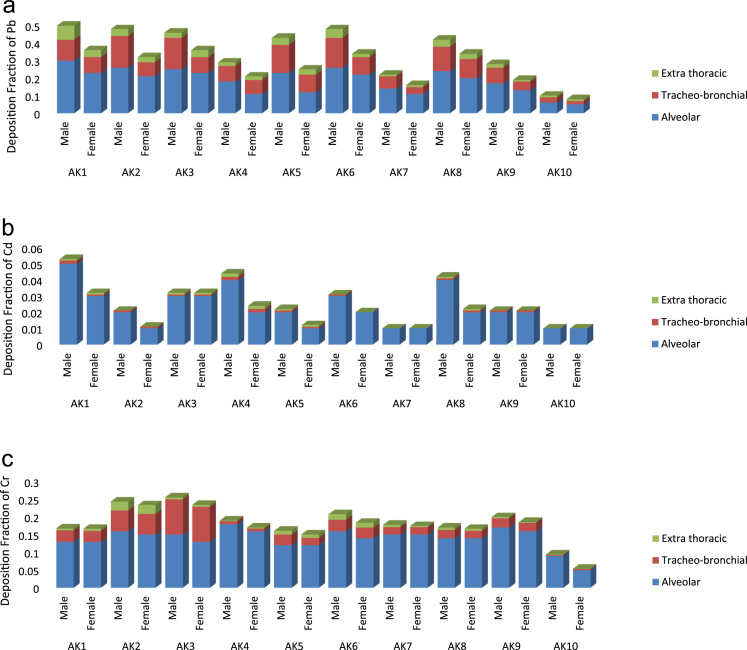
(a, b, c) Plot of deposition fraction of metals in different regions of the lung for adults.

The data sets contain the determined liable metals in various size fractions of ambient particulate matter in Akure suburbs. The data were collected in ten (10) selected locations of different air quality in Akure (Fig. 1). The coordinate and elevation of each location were determined with the aid of global positioning system (GPSMAP 78) (Table 1). All the sampling locations were impacted by anthropogenic activities. Hence, the air quality in such environment is polluted. Data on measured inhalable and respirable fractions of ambient particulate matters at the various locations are presented in Table 1. Also bioavailability index of metals determined to know the amount of soluble fraction in neutral lung are presented in Fig. 2. Statistical and numerical analyses were further carried out on data sets for further exploration; to determine metals particles dose and deposition in different regions of the lung. The analyses indicate that the highest dose and deposition of metals (Pb, Cd and Cr) occur in the alveolar region of the lung of adults (male and female) in the area (Table 3 and Fig. 3).

**Table 1:** Coordinate, Total suspended and Respirable particulate matter concentration (µg/m^3^) in studied sites of Akure suburbs.

*Sampling location*	*Coordinate*	*Elevation (m)*	*TSP (µg/m*^*3*^*)*	*Respirable fraction (µg/m*^*3*^*)*	*Inhalable fraction (µg/m*^*3*^*)*
			*Mean* ± SD	*Mean* ± SD	*Mean* ± SD
AK1	N07^o^ 17^’^ 06.2^”^	*357*	*66.47* ± 12.09	*31.16 ± 17.29*	*26.06 ± 10.29*
	E005^o^ 10^’^ 34.0^”^				
					
AK2	N07^o^ 14^’^ 25.3^”^	*374*	*64.14* ± 17.4	*26.09* ± *9.21*	*19.69* ± *8.49*
	E005^o^ 11^’^ 15.0^”^				
					
AK3	N07^o^ 15^’^ 12.7^”^	*363*	*75.47* ± *28.30*	*36.24* ± *16.22*	*30.17* ± *17.05*
	E005^o^ 11^’^ 43.8^”^				
					
AK4	N07^o^ 14^’^ 55.1^”^	*367*	*62.11* ± *.16.33*	*37.09* ± *12.71*	*32.54* ± *44.04*
	E005^o^ 12^’^ 54.4^”^				
					
AK5	N07^o^ 49^’^ 47.2^”^	*349*	*52.91* ± 14.30	*24.11* ± 1*6.38*	*21.92* ± *19.37*
	E005^o^ 09^’^ 37.9^”^				
					
AK6	N07^o^ 46^’^ 42.1^”^	*371*	*43.85 ± 12.10*	*22.41 ± 9.04*	*19.31 ± 11.9*
	E005^o^ 09^’^ 35.7				
					
AK7	N07^o^ 15^’^ 57.2^”^	*351*	*41.09 ± 10.15*	*20.95 ± 8.08*	*20.06 ± 9.05*
	E005^o^ 10^’^ 30.9				
					
AK8	N07^o^ 14^’^ 87.7^”^	*359*	*53.88 ± 11.30*	*24.43 ± 10.54*	*23.41 ± 11.36*
	E005^o^ 09^’^ 42.6				
					
AK9	N07^o^ 15^’^ 37.1^”^	*362*	*53.24 ± 17.73*	*21,48 ± 11.22*	*20.58 ± 9.41*
	E005^o^ 10^’^ 37.6				
					
AK10	N07^o^ 13^’^ 47.6^”^	*344*	*21.78 ± 6.22*	*16.94 ± 7.68*	*8.89 ± 4.99*
	E005^o^ 09^’^ 37.9^”^

## 2 Experimental design, materials and methods

Metals distributions in different size fractions of PM are of great importance to environmentalist, due to their negative contributions to human health. Emerging research is providing evidence that the metallic particles may be more dangerous than other PM components, even at the low concentrations found in ambient air. Exposure to metals in the air is capable of causing a myriad of human health effects, ranging from cardiovascular and pulmonary inflammation to cancer and damage of vital organs [1], [2]. In toxicological study, the potential health risks of individual elements bound to inhaled particulate matter depend on particle size, inhalability, bioavailability/bioaccessibility, exposure dose and deposition/ retention in respiratory tract [3], [4]. Recently, it was emphasized that bio -toxicities of trace metals depend not only on the concentration as expressed by total amount, but also on their geochemical fractions and bioavailability [4], [5]. Basic knowledge about the particle size distribution, bioavailability exposure dose and deposition of inhaled metals are crucial in evaluation of the risks associated with particulate matters exposure.

### 2.1 Study area

Akure is the capital of Ondo State which is located in the geographical coordinate of 7°10′ 0′′ N and 5°15′ 0′′ E, with average elevation of 353 m [6]. The city situated in the rainforest zone of South Western part of Nigeria with rainfall ranges from 0 to 11.59 mm. The peak rainfall months are between August and September but there are minimal rains in the months of July, October and November. According to population census of 2006, Akure has about 484,798 inhabitants [7], and by now should be close to 1 million.

### 2.2 Materials and methods

Ambient suspended particulate (TSP) samples were collected on a pre-weighed 2.5 cm Whatman glass fiber filter with pore size of 1.0 µ using a portable SKC Air check XRC 500 high volume gravimetric sampler model 210–5000, as previously described by Ediabonya et al. [8]. The respirable foam was affixed to 25 mm diameter filter for inhalable dust sampling with a flexible sample head to determine the respirable particle. The filter and cassette rear were pre-weighed to determine the initial respirable dust, while the filter, foam and whole cassette together were pre-weighed to determine the initial inhalable. After sampling, the filter, foam, with the whole cassette together were re-weighed to determine the inhalable fractions. The respirable fraction was determined by weighing the cassette rear and the filter only. These particles were collected at a flow rate of 2 L per minute of air volume for eight hours and the sampler was placed between heights of 1.5–2 m to reflect the breathing zone of man. A total of 15 samples were collected in each site for this study during dry season months between November, 2016 and March, 2017. At the end of each sampling, the filters were kept in the filter case and transported to laboratory for analysis. The concentrations of dust collected in mg/m^3^ were determined from the changes in weight of the filter (before and after sampling) divided by the volume of air sampled.

(1) $$C\;=\;\frac{\mathrm{Final\backslash weight}\;-\;\mathrm{Initial\backslash weight}\left(\mathrm{µg}\right)\times 1000}{\mathrm{Flow\backslash rate}\left(\frac{L}{\min}\right)\times \mathrm{time}(\min)}$$

For metal fractions extractable in stimulated neutral lung (bioavailability) data, the loaded filter sample was placed into a 15-ml polypropylene centrifuge tube with 10 ml of 0.01 M ammonium acetate solution at pH 7 to stimulate neutral lung environment. The tube was then made airtight and immersed for 2 h in a shaking water bath at 37°C (1-h shaking followed by 1 h still). The obtained extractants were cooled to room temperature, then centrifuged for 20 min at 3500 rpm and separated for element determination. The same filter containing the remaining non-soluble sample fraction was then digested ultrasonically using HF–HNO_3_ in the ratio of 3:1 in a hot water bath at 60°C for 6 days, with two 30-min ultra- sonication intervals.

### 2.3 Statistical and Numerical analysis

The descriptive statistics (mean value and standard deviation.) for the determined metal concentrations in inhalable, respirable and total particulate matters are presented in Table 2. The analysis was carried out using IBM SPSS statistics 20 computer software. Meanwhile, nasal inhalability is calculated using the logistic function under low wind speed (< 1ms-1) (Eq. 2) as proposed by Menache et al. [9] and the amount of particles which was actually taken across the cell membrane through inhalation pathway (bioavailability) were estimated. The data obtained are presented in Fig. 3. Finally, we employed the Multiple-path particle deposition (MPPD) mathematical model [10] to estimate deposition of elements (Pb, Cd and Cr) in different regionals of the lung (alveolar, trachea-bronchial and extra thoracic) in adults (male and female). The MPPD version 2.1 software was downloaded via http://www.ara.com/products/mppd.htm. The calculation was restricted to these metals due to their high bioavailability in this study.

(2) $$\mathrm{Inhalability}\;=\;1-[{1\;+{\;e}^{\left(13.56+0.4343\right)}X\;\log\;\mathrm{dae}\;-\;4.88)]}^{-1}$$

Where, dae(µm) is the aerodynamic particle diameter

**Table 2:** Mean metal Concentrations (µg m^-3^) in different particle sizes of ambient particulate matters in Akure suburbs.

Metal *(n=6)*	Pb(µg m^-3^) Mean ± SD		Zn(µg m^-3^) Mean ± SD		Cd (µg m^-3^) Mean ± SD		Cu (µg m^-3^) Mean ± SD		Ni (µg m^-3^) Mean ± SD		Mn(µg m^-3^) Mean ± SD		Cr(µg m^-3^) Mean ± SD		Fe(µgm^-3^) Mean ± SD	
	Inhalable	Respirable	Inhalable	Respirable	Inhalable	Respirable	Inhalable	Respirable	Inhalable	Respirable	Inhalable	Respirable	Inhalable	Respirable	Inhalable	Respirable
AK1	2.6 ± 1.4	2.3 ± 1.2	4.1 ± 1.3	3.0 ± 1.5	1.9 ± 2.0	1.1 ± 1.1	3.6 ± 0.3	3.2 ± 1.4	0.9 ± 0.1	0.5 ± 0.1	2.3 ± 0.6	2.1 ± 0.7	2.0 ± 0.6	1.8 ± 0.3	3.4 ± 1.2	3.1 ± 0.7
AK2	1.8 ± 0.3	1.6 ± 0.4	3.9 ± 0.3	2.9 ± 0.9	1.7 ± 1.6	1.3 ± 1.0	2.5 ± 0.9	2.8 ± 1.5	0.4 ± 0.2	0.3 ± 0.2	1.8 ± 0.4	1.5 ± 0.3	2.8 ± 0.1	1.1 ± 0.6	2.6 ± 1.1	1.4 ± 0.4
AK3	3.6 ± 0.4	3.5 ± 0.8	4.6 ± 1.4	4.2 ± 1.1	2.0 ± 1.6	1.2 ± 0.6	3.8 ± 0.7	4.3 ± 1.2	0.8 ± 0.6	0.6 ± 0.3	3.5 ± 1.1	1.4 ± 0.4	3.1 ± 0.9	1.4 ± 0.4	1.6 ± 0.5	1.4 ± 0.8
AK4	3.1 ± 2.2	2.8 ± 1.3	3.3 ± 1.9	2.8 ± 0.2	2.0 ± 0.5	1.2 ± 0.4	3.5 ± 0.4	4.5 ± 1.8	0.8 ± 0.6	0.7 ± 0.3	2.8 ± 1.6	1.3 ± 0.8	3.0 ± 0.2	1.9 ± 0.7	1.8 ± 1.2	1.2 ± 0.1
AK5	2.7 ± 1.6	1.7 ± 0.6	2.7 ± 0.9	2.3 ± 0.6	1.4 ± 0.9	1.1 ± 0.1	2.6 ± 0.9	2.8 ± 0.7	0.6 ± 0.2	0.4 ± 0.2	2.7 ± 1.4	1.4 ± 0.6	2.3 ± 0.5	1.6 ± 0.5	2.4 ± 0.5	1.8 ± 0.7
AK6	1.8 ± 0.5	1.7 ± 0.5	2.1 ± 1.1	1.9 ± 0.6	2.3 ± 1.3	1.3 ± 0.9	3.5 ± 1.5	2.6 ± 0.9	0.8 ± 0.3	0.7 ± 0.3	1.6 ± 1.1	1.3 ± 0.8	2.0 ± 0.3	1.8 ± 0.7	2.6 ± 0.3	1.1 ± 0.3
AK7	0.7 ± 0.4	0.6 ± 0.3	2.4 ± 1.3	1.5 ± 1.0	2.1 ± 0.5	1.3 ± 0.8	2.5 ± 1.3	3.0 ± 1.6	0.7 ± 0.3	0.5 ± 0.2	0.7 ± 0.3	1.3 ± 0.3	2.2 ± 0.3	1.6 ± 0.4	2.3 ± 0.8	1.9 ± 0.4
AK8	2.8 ± 1.6	2.5 ± 1.1	3.1 ± 0.8	2.4 ± 0.4	1.9 ± 0.7	1.6 ± 0.3	2.4 ± 1.4	1.8 ± 0.8	0.6 ± 0.2	0.7 ± 0.3	2.6 ± 1.5	1.5 ± 0.9	1.8 ± 0.2	1.4 ± 0.6	1.8 ± 0.5	1.2 ± 0.6
AK9	1.9 ± 0.8	1.6 ± 0.9	2.4 ± 0.6	1.7 ± 0.2	2.0 ± 0.8	1.2 ± 0.7	2.4 ± 0.9	1.7 ± 0.9	0.9 ± 0.4	0.9 ± 0.4	1.5 ± 0.4	1.3 ± 0.3	1.7 ± 0.5	1.4 ± 0.5	1.8 ± 0.4	1.5 ± 0.3
AK10	0.6 ± 0.1	0.7 ± 0.3	0.8 ± 0.2	0.9 ± 0.3	1.1 ± 0.3	0.9 ± 0.5	1.1 ± 0.6	0.7 ± 0.3	0.2 ± 0.1	0.1 ± 0.1	0.9 ± 0.3	0.8 ± 0.4	0.2 ± 0.1	0.3 ± 0.2	0.6 ± 0.3	0.1 ± 0.1

**Table 3:** Estimated doses of metal particles in different regions of lung at various studied sites of Akure.

*Site*	*Respiratory tracts*	*Dose (µgd*^*-1*^*)*							
		Pb	Zn	Cd	Cu	Ni	Mn	Cr	Fe
AK1	Alveolar	3.23	0.19	1.11	0.48	0.62	0.12	4.09	0.05
	Trachea-bronchial	2.81	0.16	0.61	0.32	0.43	0.06	2.94	0.04
	Extra thoracic	1.62	0.03	0.39	0.10	0.30	0.02	1.52	0.01
									
AK2	Alveolar	3.66	0.63	1.67	0.50	0.82	0.28	5.12	0.06
	Trachea-bronchial	2.95	0.28	1.01	0.39	0.54	0.09	3.36	0.04
	Extra thoracic	2.34	0.22	0.89	0.12	0.47	0.02	1.86	0.02
									
AK3	Alveolar	3.68	0.47	1.92	0.74	0.80	0.18	4.33	0.09
	Trachea-bronchial	2.44	0.27	1.43	0.67	0.72	0.07	2.90	0.08
	Extra thoracic	2.00	0.23	1.28	0.45	0.53	0.04	1.47	0.06
									
AK4	Alveolar	3.29	0.25	1.34	0.65	0.47	0.10	2.56	0.09
	Trachea-bronchial	1.36	0.20	1.26	0.51	0.44	0.08	1.17	0.06
	Extra thoracic	1.12	0.13	0.22	0.4	0.13	0.05	1.01	0.03
									
ArK5	Alveolar	2.15	0.35	1.28	0.71	0.63	0.13	3.28	0.07
	Trachea-bronchial	1.86	0.29	0.63	0.49	0.41	0.08	1.48	0.05
	Extra thoracic	1.41	0.24	0.17	0.20	0.22	0.03	1.34	0.03
									
AK6	Alveolar	4.33	0.68	1.01	0.52	0.70	0.12	2.80	0.08
	Trachea-bronchial	2.64	0.52	0.77	0.38	0.52	0.09	1.67	0.05
	Extra thoracic	2.05	0.21	0.42	0.29	0.31	0.05	1.21	0.03
									
AK7	Alveolar	3.38	0.55	1.16	0.64	0.50	0.13	2.33	0.09
	Trachea-bronchial	2.54	0.22	0.85	0.38	0.37	0.09	1.66	0.04
	Extra thoracic	1.96	0.19	0.31	0.22	0.24	0.03	1.09	0.03
									
AK8	Alveolar	3.29	0.23	1.21	0.53	0.97	0.15	2.58	0.10
	Trachea-bronchial	3.03	0.11	0.98	0.33	0.55	0.08	1.18	0.05
	Extra thoracic	2.74	0.05	0.47	0.17	0.23	0.03	1.06	0.02
									
AK9	Alveolar	3.98	0.40	1.23	0.67	0.88	0.14	2.37	0.09
	Trachea-bronchial	2.35	0.23	1.04	0.44	0.63	0.05	1.30	0.06
	Extra thoracic	1.99	0.16	0.94	0.25	0.47	0.01	1.14	0.03
									
AK10	Alveolar	1.12	0.20	1.02	0.20	0.10	0.07	1.01	0.03
	Trachea-bronchial	0.62	0.09	0.06	0.11	0.05	0.01	0.04	0.01
	Extra thoracic	0.07	0.05	0.06	0.08	0.04	0.01	0.00	0.01

The mass of particulate matter Crt(µg) that enter the respiratory tracts was calculated based on the following Eq. (3).

(3) Crt(µg)=VxDFxC

*Where,* V= minute ventilation (m^3^ min^-1^)-set at different exercises, according to ICRP [11], C = concentration of particulate matter present in air

The daily dose of inhaled metal (µg d^-1^) was calculated as follows;

(4) $$\mathrm{Dose}\left(\frac{µg}{d}\right)=\mathrm{DF}\times C\times t\times \mathrm{IR}$$

Where, DF= deposited fraction of particle in different regions of respiratory tract, C = concentration of metal inhaled per volume of air (particle cm^−3^), t = the exposure time (hours), IR.
